# *Porphyromonas gingivalis* lipopolysaccharide induces cognitive dysfunction, mediated by neuronal inflammation via activation of the TLR4 signaling pathway in C57BL/6 mice

**DOI:** 10.1186/s12974-017-1052-x

**Published:** 2018-02-09

**Authors:** Jing Zhang, Chunbo Yu, Xuan Zhang, Huiwen Chen, Jiachen Dong, Weili Lu, Zhongchen Song, Wei Zhou

**Affiliations:** 1 Department of Periodontology, Ninth People’s Hospital, Shanghai Jiao Tong University School of Medicine, Shanghai, China; 2 Ninth People’s Hospital, Shanghai Jiao Tong University School of Medicine, Shanghai, China; 3 Department of Pharmacy, Ninth People’s Hospital, Shanghai Jiao Tong University School of Medicine, Shanghai, China; 4 Laboratory of Oral Microbiota and Systemic Diseases, Shanghai Research Institute of Stomatology, Ninth People’s Hospital, Shanghai Jiao Tong University School of Medicine, Shanghai, China; 5 Shanghai Key Laboratory of Stomatology & Shanghai Research Institute of Stomatology, National Clinical Research Center of Stomatology, Shanghai, China

**Keywords:** *Porphyromonas gingivalis*, Lipopolysaccharide, Cognition, Neuroinflammation, TLR4

## Abstract

**Background:**

*Porphyromonas gingivalis* lipopolysaccharide (*P. gingivalis*-LPS) is one of the major pathogenic factors of chronic periodontitis (CP). Few reports on the correlation between *P. gingivalis*-LPS and cognitive function exist. Thus, the present study aimed to investigate the effects of *P. gingivalis*-LPS on cognitive function and the associated underlying mechanism in C57BL/6 mice.

**Methods:**

The C57BL/6 mice were injected with *P. gingivalis*-LPS (5 mg kg^−1^) either with or without Toll-like receptor 4 (TLR4) inhibitor (TAK-242, 5 mg kg^−1^). After 7 days, behavioral alterations were assessed with the open field test (OFT), Morris water maze (MWM) test, and passive avoidance test (PAT). The activation of astrocytes and microglia in the cerebral cortex and hippocampus of mice was observed by immunohistochemistry. The expression of inflammatory cytokines (TNF-α, IL-1β, IL-6, and IL-8), TLRs (TLR2, TLR3, and TLR4), and CD14 and the activation of the NF-κB signaling pathway (IRAK1, p65, and p-p65) in the cerebral cortex of the mice were evaluated by RT-PCR, ELISA, and western blot.

**Results:**

The OFT showed that *P. gingivalis*-LPS did not affect the initiative and activity of mice. Administration of *P. gingivalis*-LPS significantly impaired spatial learning and memory during the MWM test and attenuated the ability of passive avoidance learning during the PAT. Both astrocytes and microglia were activated in the cortex and hippocampus. The messenger RNA (mRNA) and protein expression of inflammatory cytokines (TNF-α, IL-1β, IL-6, and IL-8) was upregulated by *P. gingivalis*-LPS in the cortex. In addition, the TLR4/NF-κB signaling pathway was activated (TLR4, CD14, IRAK1, and p-p65). These effects were effectively alleviated by TAK-242.

**Conclusions:**

Administration of *P. gingivalis*-LPS can lead to learning and memory impairment in C57BL/6 mice. This impairment is mediated by activation of the TLR4 signaling pathway. Our study suggests that *P. gingivalis*-LPS-induced neuroinflammation plays an important role in cognitive impairment. It also reveals that endotoxins of periodontal pathogens could represent a risk factor for cognitive disorders.

## Background

Periodontitis is one of the most common oral chronic inflammatory diseases. It can lead to systemic inflammatory responses and other diseases, including cardiovascular disease and respiratory disease, among others [1–3]. In recent years, studies have supported a high correlation between chronic periodontitis (CP) and dementia [4, 5]. A relatively large number of clinical observations and epidemiological evidence have shown that periodontitis is a risk factor for disorders of the central nervous system (CNS) [4–7].

*P. gingivalis* has been recognized as one of the most common Gram-negative anaerobic bacteria in CP. The lipopolysaccharide (LPS) located in the outer membrane of Gram-negative bacteria is the main pathogenic factor of *P. gingivalis*. The *P. gingivalis* lipopolysaccharide (*P. gingivalis*-LPS) can continuously stimulate immune cells of the main host, specifically monocytes (MO)/macrophages (MΦ). This stimulation results in the release of a large number of bioactive substances, such as lysosomal enzymes, cytokines, reactive oxygen species, and nitric oxide, and leads to cell damage, apoptosis, and ultimately, inflammation [8, 9].

Although *P. gingivalis*-LPS and *Escherichia coli* lipopolysaccharide (*E. coli*-LPS) are both derived from Gram-negative bacteria, they differ in structure and function. The two types of LPS differentially modulate Toll-like receptor 2 (TLR2), TLR4, and CD14 surface expression, as well as primary and secondary cytokine responses [10, 11]. The *E. coli*-LPS can reportedly lead to cognitive dysfunction in mice [12, 13], which activates the TLR4/NF-κB signaling pathway [14]. However, few studies have focused on the relationship between *P. gingivalis*-LPS and cognitive function. Poole et al. observed *P. gingivalis*-LPS in the brain tissue of patients with Alzheimer’s disease on autopsy, suggesting a possible relationship between *P. gingivalis*-LPS and cognitive dysfunction [15]. However, to our knowledge, no studies report on the effects of *P. gingivalis*-LPS on cognitive function in vivo and the underlying mechanism.

In the present study, we attempted to observe the effects of *P. gingivalis*-LPS on cognitive function in C57BL/6 mice, using various tasks in the open field test (OFT), Morris water maze (MWM) test, and passive avoidance test (PAT). The activation of microglia and astrocytes in the cerebral cortex and hippocampus was observed by immunohistochemistry. To further examine the underlying associated mechanism, the relative expression of inflammatory cytokines in the cerebral cortex was assessed by RT-PCR and ELISA. Furthermore, the activation of the TLR4/NF-κB signaling pathway was assessed by western blot analysis.

## Methods

Specific pathogen-free (SPF), 8-week-old, male C57BL/6 mice, weighing 20–25 g each, were selected. Animals were placed in a temperature- and humidity-controlled room under a 12-h light/dark cycle, with free access to food and water. All experimental protocols were approved by the ethical committee of the Animal Care and Experimental Committee of Shanghai Jiao Tong University School of Medicine and were performed according to the guidelines from the EU Directive 2010/63/EU.

**Fig. 1:**
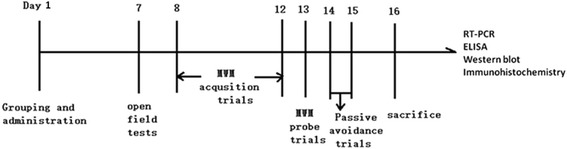
Experimental design. *P.gingivalis*-LPS (5 mg kg^−1^, i.p.) was administered in the *P.gingivalis*-LPS group and *P.gingivalis*-LPS plus TAK group. Seven days after *P.gingivalis*-LPS administration, cognitive function was assessed by the open field test (OFT), Morris water maze (MWM) test, and passive avoidance test (PAT). The underlying mechanism was further detected by RT-PCR, ELISA, western blot, and immunohistochemistry

Sixty mice were randomly divided into five groups. The *P. gingivalis*-LPS group, *E. coli*-LPS group, TAK group, and *P. gingivalis*-LPS plus TAK group were all administered *P. gingivalis*-LPS (5 mg kg^−1^, i.p.) and TAK-242 (5 mg kg^−1^, i.p., 1 h before administration of *P. gingivalis*-LPS). The control group was given an equivalent volume of saline. Seven days after administration of the treatment, behavioral tests were performed to evaluate the cognitive function of the mice after recovery of motor activity. The study design is presented in Fig. 1.

The *P. gingivalis*-LPS and TAK-242 were purchased from InvivoGen (San Diego, CA, USA), and the *E. coli*-LPS (*Escherichia coli* serotype 0111:B4) was purchased from Sigma (St. Louis, MO, USA). Drugs were dissolved according to the manufacturer’s instructions.

### Open field test

The OFT was used to assess spontaneous activity, anxiety-like behavior, and emotional change in the animals [16]. When placed in an open field for the first time, mice tend to remain along the periphery of the apparatus because of fear. Therefore, anxiety-like behavior could be assessed, based on the extent of exploratory behavior observed. The open field in the present study consisted of a rectangular arena (278 × 236 mm), enclosed by a white wall, 30 cm in height. The test was initiated by gently placing a single mouse in the middle of the arena, allowing the animal to move freely for 5 min, while being recorded.

### Morris water maze test

The MWM test is used to test spatial learning and memory [17]. The MWM equipment (Datum Mobile, Minhang, Shanghai, China) consisted of a circular pool with a diameter of 120 cm and a depth of 50 cm. The pool was filled with water until a platform (9 cm in diameter) in the pool was submerged 1 cm below the surface. The water temperature was controlled to remain with a range equivalent to that of room temperature (22 ± 1 °C). A video analysis system was used to monitor and record the swimming activity of the mice.

The mice should have learned to use the visual tips around the pool to find the hidden platforms within 90 s. If a mouse could not find the platform within 90 s, it was guided to the platform and allowed to stay there for 30 s. Each mouse was trained four times a day with 30 s of rest per training interval. The probe trial was carried out on the sixth day. The platform was removed from the pool, and the mice were then placed into the water at the two quadrants furthest from the platform used on days 1–5. Each mouse was allowed to navigate for 60 s.

### Passive avoidance test

The PAT was used to evaluate passive avoidance learning in mice [18]. Mice tend to prefer darkness; thus, the device was divided by a retractable door into two compartments: a brightly lit compartment and a dark compartment. Upon completely entering the dark compartment, the animal received an electric shock (0.6 mA, 2 s).

The first day of the study was designated training day. Each mouse was placed in the light compartment, and 10 s later, the door was opened. After 5 min, the mouse was removed. These actions were repeated for each mouse. When the mouse entered the dark compartment, it immediately received the electric shock. The time taken (latency) for the mouse to enter the dark compartment was recorded. A retention test was again conducted 24 h later. The latency to re-enter the dark compartment and the number of electrical shocks (error times) within 5 min were recorded.

### RNA extraction and RT-PCR analysis

The tissues of the cortex of mice were homogenized, and RNA was isolated using the Total RNA Kit (Omega Bio-Tek, Inc., Norcross, GA, USA). The RNA was reverse transcribed into cDNA using the M-MLV reverse transcriptase kit (Invitrogen, Waltham, MA, USA). The sequences of these primers were as follows: TNF-α, 5′-GACAAGGCTGCCCCGACTACG-3′ (forward) and 5′-CTTGGGGCAGGGGCTCTTGAC-3′ (reverse); IL-1β, 5′-AAATCTCGCAGC AGCACATCAA-3′ (forward) and 5′-CCACGGGAAAGACACAGGTAGC-3′ (reverse); IL-6, 5′-AGTTGCCTTCTTGGGACTGA-3′ (forward) and 5′-CCTCC GACTTGTGAAGTGGT-3′ (reverse); IL-8, 5′-CAGCTGCCTTAACCCCATCA-3′ (forward) and 5′-CTTGAGAAGTCCATGGCGAAA-3′ (reverse); TLR2, 5′-CTC CTGAAGCTGTTGCGTT AC-3′ (forward) and 5′-TACTTTACCCAGCTCGCTC ACTAC-3′ (reverse); TLR3, 5′-CAGGATACTTGATCTCGGCCTT-3′ (forward) and 5′-TGGCCGCTGAGTTTTTGTTC-3′ (reverse); TLR4, 5′-CGCTTTCACCT CTGCCTTCACTACAG-3′ (forward) and 5′-ACACTACCACAATAACCTTCCG GCTC-3′ (reverse); CD14, 5′-TGTCGTGGGCAACAAGGGATG-3′ (forward) and 5′-AAGGTGGAGAGGGCAGGGAAGA-3′ (reverse); and actin, 5′-CATCCGTA AAGACCTCTATGCCAAC-3′ (forward) and 5′-ATGGAGCCACCGATCCACA-3′ (reverse). Subsequently, RT-PCR analyses were performed using the SYBR premix EX Taq™ (Takara, Kusatsu, Shiga, Japan) according to the manufacturer’s protocol in a Light Cycler 480 System (Roche Diagnostics, Grenzacherstrasse, Basel, Switzerland). The DNA amplification was performed as follows: the first cycle was maintained at 95 °C for 30 s, followed by 40 cycles consisting of denaturation (95 °C for 10 s), annealing (60 °C for 20 s), and extension (72 °C for 15 s). The TNF-α, IL-1β, and IL-8 were then processed using the 2^−ΔΔCt^ method, during which a single calibrated sample was compared against the gene expression of every unknown sample.

### ELISA analysis

Radioimmunoprecipitation assay (RIPA) lysis buffer (Beyotime, Beijing, China), 1% protease inhibitor cocktail (Sigma, St. Louis, MO, USA), and 1% phenylmethylsulfonyl fluoride (PMSF; Beyotime, Beijing, China) were used to homogenize samples of the cerebral cortex. Protein quantification was performed using the BCA (bicinchoninic acid) Protein Assay Kit (Thermo Scientific, Vernon Hills, IL, USA). For quantification by ELISA, commercially available ELISA kits were used according to the manufacturer’s instructions to measure levels of TNF-α, IL-6, IL-8 (UBI, Sunnyvale, CA, USA), and IL-1β (Westang, Yangpu, Shanghai, China) in the cortex.

### Western blot

The RIPA lysis buffer, in addition to 1% protease inhibitor cocktail and 1% PMSF, was used to homogenize samples of the cerebral cortex. Following the addition of sodium dodecyl sulfate (SDS) loading buffer, the samples were boiled for 5 min, and proteins were subsequently detected by western blot analysis. The proteins were transferred to a polyvinylidene difluoride (PVDF) membrane after separation. A wide range of protein markers was run in parallel to detect the molecular weight of proteins. Skimmed milk was used for membrane blockage to reduce nonspecific binding. Proteins were probed with anti-TLR4 (1:1000, ab13867; Abcam, Cambridge, UK), anti-CD14 (1:500, ab203294; Abcam), anti-IRAK1 (1:1000, no. 4504; Cell Signaling Technology, Danvers, MA, USA), anti-p65 (1:1000, no. 8242; Cell Signaling Technology), anti-phospho-p65 (1:1000, no. 3033; Cell Signaling Technology), and anti-β-actin (1:1000, abs119600; Abcam). The data were quantified using the Image Studio Lite ver. 5.2 software.

### Immunohistochemistry

Mice were anesthetized with chloral hydrate and perfused with physiological saline before sacrifice. Immediately after removal of the brain, one hemisphere was placed in 4% paraformaldehyde and left overnight at 4 °C, after which paraffin sections were prepared. The cortex of the other hemisphere was immediately removed and stored at − 80 °C for subsequent ELISA, RT-PCR, and western blot analyses.

Brain sections were incubated with 3% H_2_O_2_ in methanol for 10 min to quench endogenous peroxidase activity. They were then rinsed with phosphate-buffered saline (PBS), blocked with 10% goat serum for 30 min, and incubated overnight at 4 °C with the following primary antibodies: rabbit anti-glial fibrillary acidic protein (GFAP) (1:400, ab7260; Abcam) and goat Iba1 (1:400, ARG63338; Arigo Biolaboratories, Hsinchu City, Taiwan, China). After being washed, sections were incubated with biotinylated goat anti-rabbit or goat secondary antibody (1:200; Vector Laboratories, Burlingame, CA, USA). After being rinsed with PBS, streptavidin-labeled peroxidase was added and left for 30 min. This was followed by further rinsing, after which newly prepared 3,3′-diaminobenzidine (DAB) solution was added, and the mixture was left for the reaction to develop. The sections were dyed with hematoxylin and dipped in 1% hydrochloric acid in alcohol for differentiation. They were then washed in ammonia and stained blue, after which they were rinsed with water.

For cell counting, images were obtained with a Nikon camera. The numbers of GFAP-positive cells were determined by counting positive cells in two areas of each section in every tenth serial coronal section. At least three coronal sections were analyzed per mouse, and the average of the individual measurements was used to calculate group means [19].

### Data analysis

Data were presented as mean ± standard error of the mean (SEM). Statistical analyses were performed using repeated measures ANOVA, one-way ANOVA, and Student’s *t* test with the GraphPad Prism software. An analysis of variance was performed using Tukey’s post hoc multiple comparison test. A value of *p* < 0.05 was indicative of statistical significance.

## Results

### Effects of *P. gingivalis*-LPS on activity of the animal

**Fig. 2:**
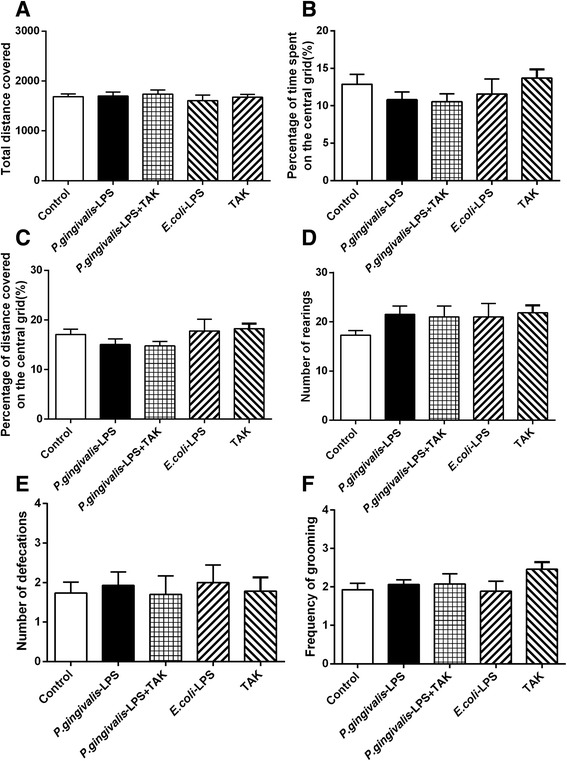
Effects of *P.gingivalis*-LPS on animal activity. The open field test (OFT) was used to evaluate the spontaneous activities of mice after 7 days of administration, including total distance covered (**a**), percentage of time spent on central grid (**b**), percentage of distance covered on central grid (**c**), number of rearings (**d**), number of defecations (**e**), and frequency of grooming (**f**). Overall, no significant differences were observed between treatment groups

As shown in Fig. 2, the behaviors of mice (total distance covered, percentage of distance covered on the central grid, percentage of time spent on the central grid, number of defecations, number of rearings, and frequency of grooming) showed no significant differences among all groups in the OFT. The behavioral performance indicated that the activity of the mice was not affected by *P. gingivalis*-LPS.

### Effects of *P. gingivalis*-LPS on spatial learning and memory of C57BL/6 mice in the MWM test

**Fig. 3:**
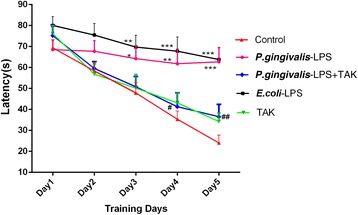
Effects of *P.gingivalis*-LPS on spatial learning of mice during the Morris water maze (MWM) test. Effects of treatment with *P.gingivalis*-LPS (5 mg kg^−1^, i.p.) alone or in combination with TAK-242 (5 mg kg^−1^, i.p.) on latency to find the platform during the acquisition phase of the MWM test. Data are presented as mean ± SEM; *p < 0.05, **p < 0.01 and ***p < 0.001, compared to the control group; ^#^*p* < 0.05 and ^##^*p* < 0.01, compared to the *P.gingivalis*-LPS group

To assess spatial learning acquisition, mice were trained in the MWM task during four trials per day for five consecutive days. In all groups, the escape latency showed a marked decline over the training days. In the *P. gingivalis*-LPS group, the escape latency was evidently longer than that in the control group at day 3 (64.25 ± 5.97 versus 47.91 ± 4.93 s, respectively), day 4 (61.81 ± 7.07 versus 35.36 ± 3.90 s, respectively), and day 5 (62.69 ± 6.73 versus 24.06 ± 3.71 s, respectively). Moreover, the *P. gingivalis*-LPS plus TAK group showed a noticeably shorter latency in comparison to the *P. gingivalis*-LPS group at day 4 (41.33 ± 6.53 versus 61.81 ± 7.07 s, respectively) and day 5 (36.54 ± 5.83 versus 62.69 ± 6.73 s, respectively). No significant differences were observed between the *P. gingivalis*-LPS group and *E. coli*-LPS group (Fig. 3). Results of the probe trial illustrate that the learning ability of mice can be impaired by *P. gingivalis*-LPS. However, this change was significantly prevented by TAK-242.

**Fig. 4:**
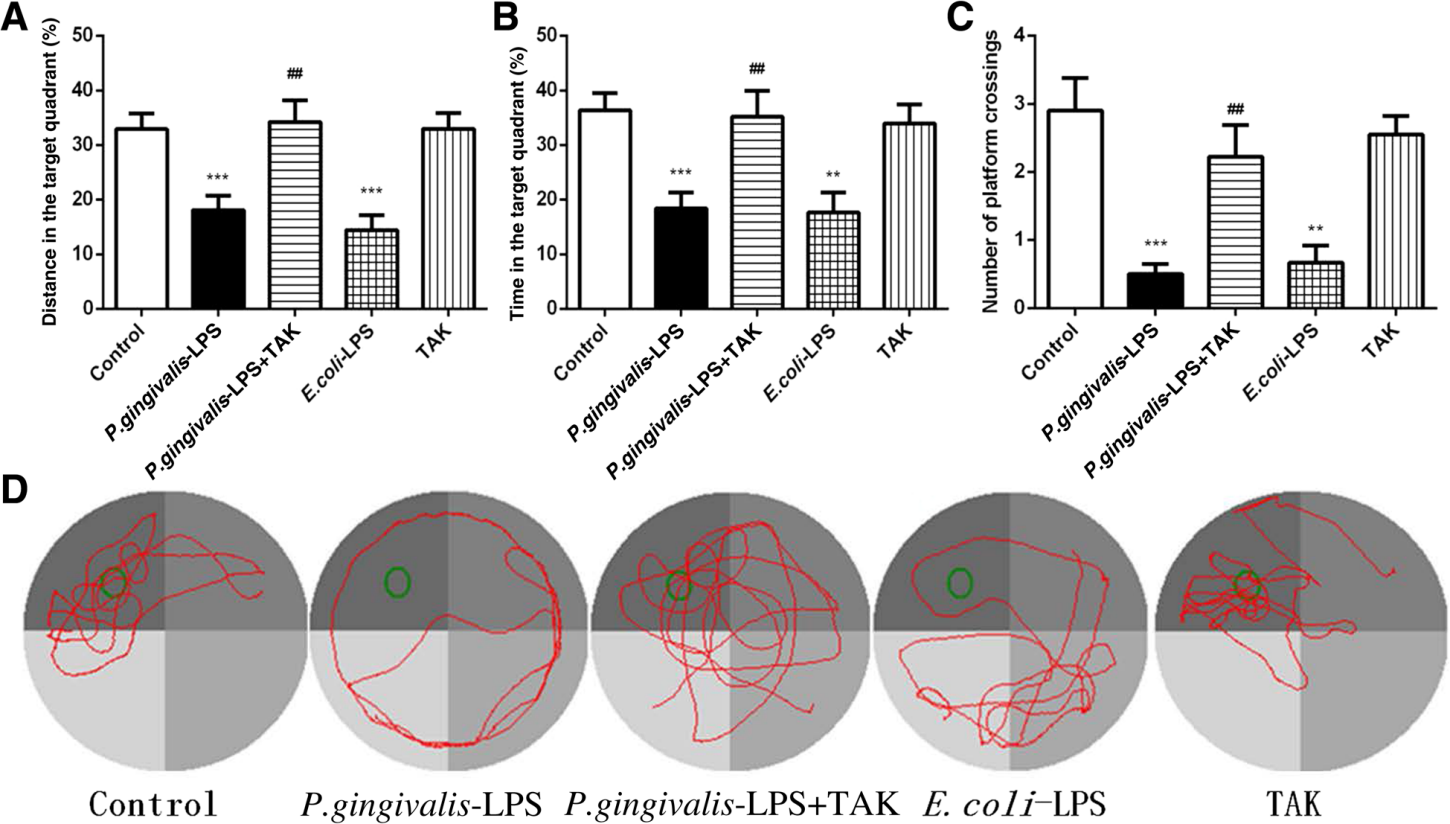
Effects of *P.gingivalis*-LPS on spatial memory and trajectories in the Morris water maze (MWM) test. The platform was removed on the sixth day, and the distance traveled in the target quadrant was compared between the groups. The following parameters were assessed: percentage of the distance covered in the target quadrant (**a**), percentage of time spent in the target quadrant (**b**), and number of platform crossings in the target quadrant (**c**). The percentage of distance, percentage of time, and number of platform crossings in the target quadrant were significantly reduced by *P.gingivalis*-LPS. The typical trajectories of the *P.gingivalis*-LPS group approximated the arc of a circle, without any crossings over the original platform (**d**). Data are presented as mean ± SEM; **p < 0.01, ***p < 0.001, compared to the control group; ^##^*p* < 0.01, compared to the *P.gingivalis*-LPS group

During the spatial probe test, the number of platform crossings was significantly reduced in the *P. gingivalis*-LPS group, in comparison to the control group (Fig. 4c). Furthermore, the *P. gingivalis*-LPS group traveled a shorter distance (18.05 ± 2.66 versus 32.95 ± 2.85% for the control group) and spent less time (18.36 ± 2.91 versus 36.39 ± 3.12% for the control group) in the target quadrant (Fig. 4a, b). In contrast, mice in the *P. gingivalis*-LPS plus TAK group traveled a longer distance (34.21 ± 4.02 versus 18.05 ± 2.66% for the *P. gingivalis*-LPS group) and spent more time (35.24 ± 4.75 versus 18.36 ± 2.91% for the *P. gingivalis*-LPS group) in the target quadrant than those in the *P. gingivalis*-LPS group. No significant differences were found between the *P. gingivalis*-LPS group and *E. coli*-LPS group (Fig. 4). Results of the probe trial illustrate that the learning ability of mice can be impaired by *P. gingivalis*-LPS. These effects, however, were significantly prevented by TAK-242.

### Effects of *P. gingivalis*-LPS on passive avoidance learning in mice

**Fig. 5:**
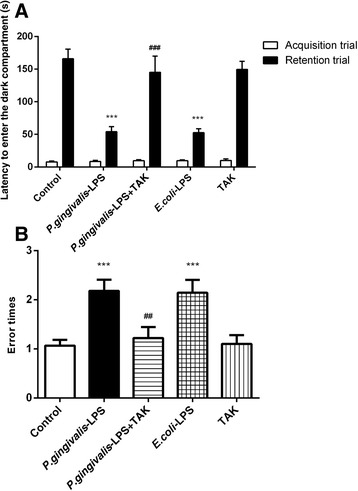
Effects of *P.gingivalis*-LPS on response and memory of mice in the passive avoidance test (PAT). The PAT was used to evaluate passive avoidance learning in mice following the Morris water maze (MWM) test. The following parameters were determined: latency to enter the dark compartment (**a**) and error times to enter the dark compartment (**b**). Administration of *P.gingivalis*-LPS reduced the latency and increased error times to enter the dark compartment. Data are presented as mean ± SEM; ***p < 0.001, compared to the control group; ^##^*p* < 0.01 and  ^###^*p* < 0.001, compared to the *P.gingivalis*-LPS group

During the first day of training, no significant differences were observed in the latency of each group to enter the dark compartment. After the electric shock was given, the memory was retained the following day. As shown in Fig. 5, the *P. gingivalis*-LPS group showed a reduced latency to enter the dark compartment as compared with the control group (54.00 ± 7.67 versus 165.90 ± 14.68 s for the control group) and this was significantly prevented by TAK-242 (134.20 ± 24.42 s). Furthermore, The *P. gingivalis*-LPS group showed more error times to enter the dark compartment than the control group (2.18 ± 0.23 versus 1.07 ± 0.12 for the control group) and this was significantly prevented by TAK-242 (1.22 ± 0.22). No significant differences were observed between the *P. gingivalis*-LPS group and *E. coli*-LPS group. The PAT results indicated that passive avoidance learning in mice could be impaired by *P. gingivalis*-LPS. These effects, however, were significantly prevented by TAK-242.

### Effects of *P. gingivalis*-LPS on microglia and astrocytes in the cortex and hippocampus

**Fig. 6:**
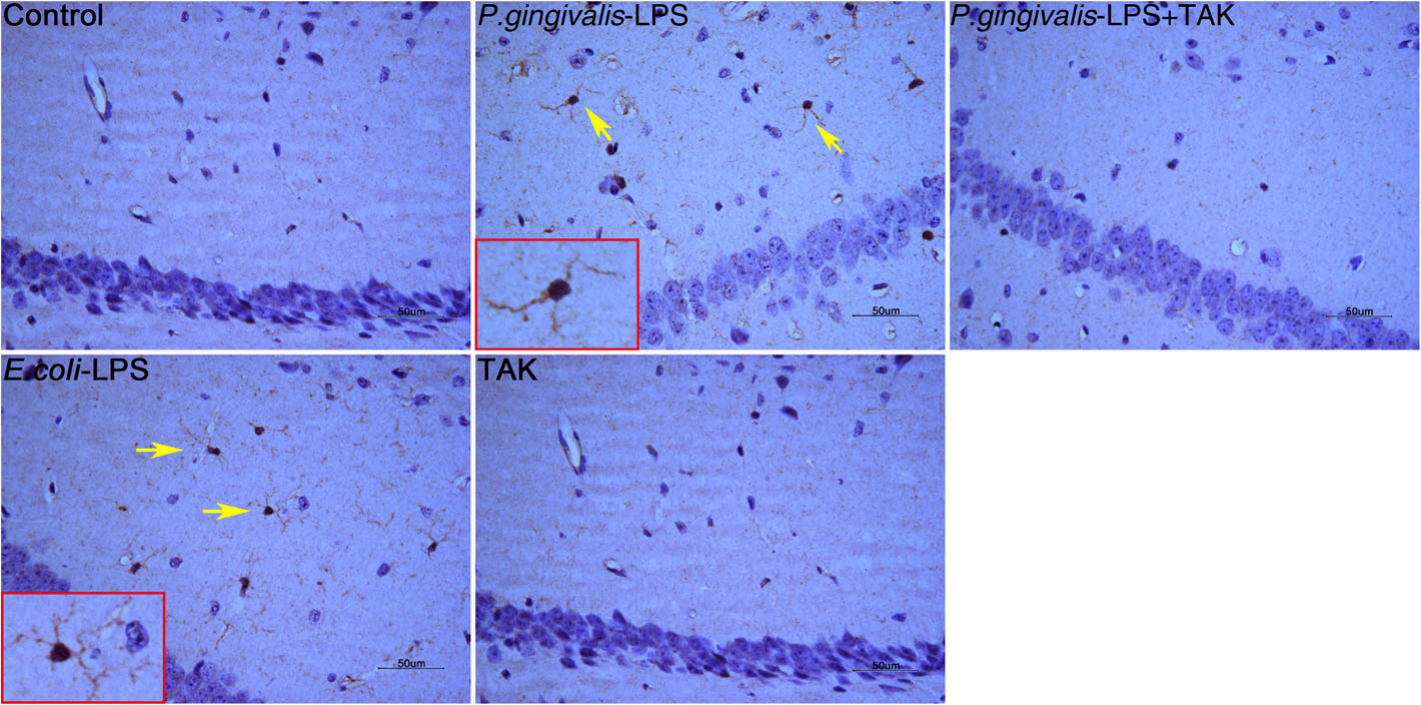
Effects of *P.gingivalis*-LPS on microglia in the hippocampus. Histopathological analysis of brain sections was performed using immunohistochemistry. Microglia were visualized with ionized calcium-binding adaptor molecule 1 (Iba1) (arrows). Activated microglia with irregular protrusions were observed in the *P.gingivalis*-LPS group (bar = 50 μm)

**Fig. 7:**
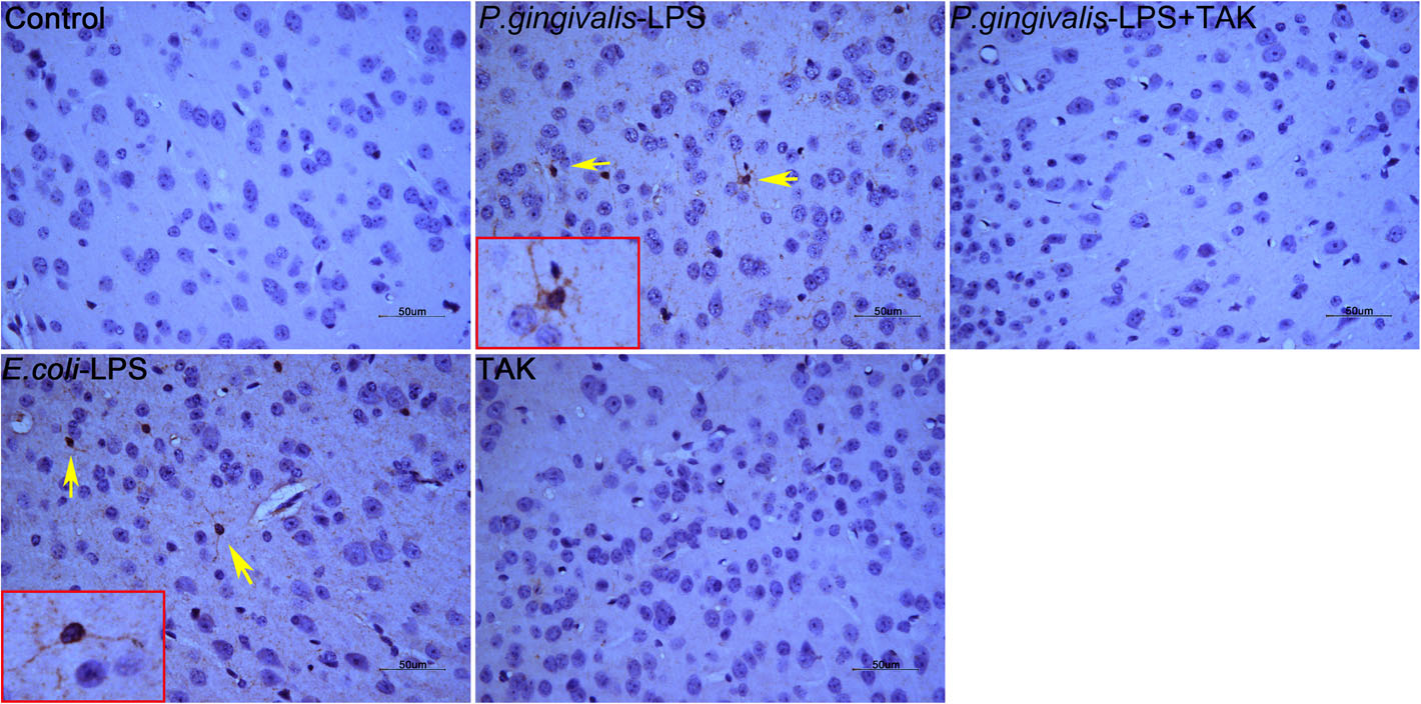
Effects of *P.gingivalis*-LPS on microglia in the cortex. Histopathological analysis of brain sections was performed using immunohistochemistry. Microglia were visualized with ionized calcium-binding adaptor molecule 1 (Iba1) (arrows). Activated microglia with irregular protrusions were observed in the *P.gingivalis*-LPS group (bar = 50 μm)

The activated microglia, characterized by irregular protrusions and increased volume of the cell bodies, were positively stained with ionized calcium-binding adaptor molecule 1 (Iba1). Activated microglia were observed in the hippocampus and cortex of the *P. gingivalis*-LPS group and *E. coli*-LPS group, whereas activated microglia were rarely observed in the control group (Figs. 6 and 7).

**Fig. 8:**
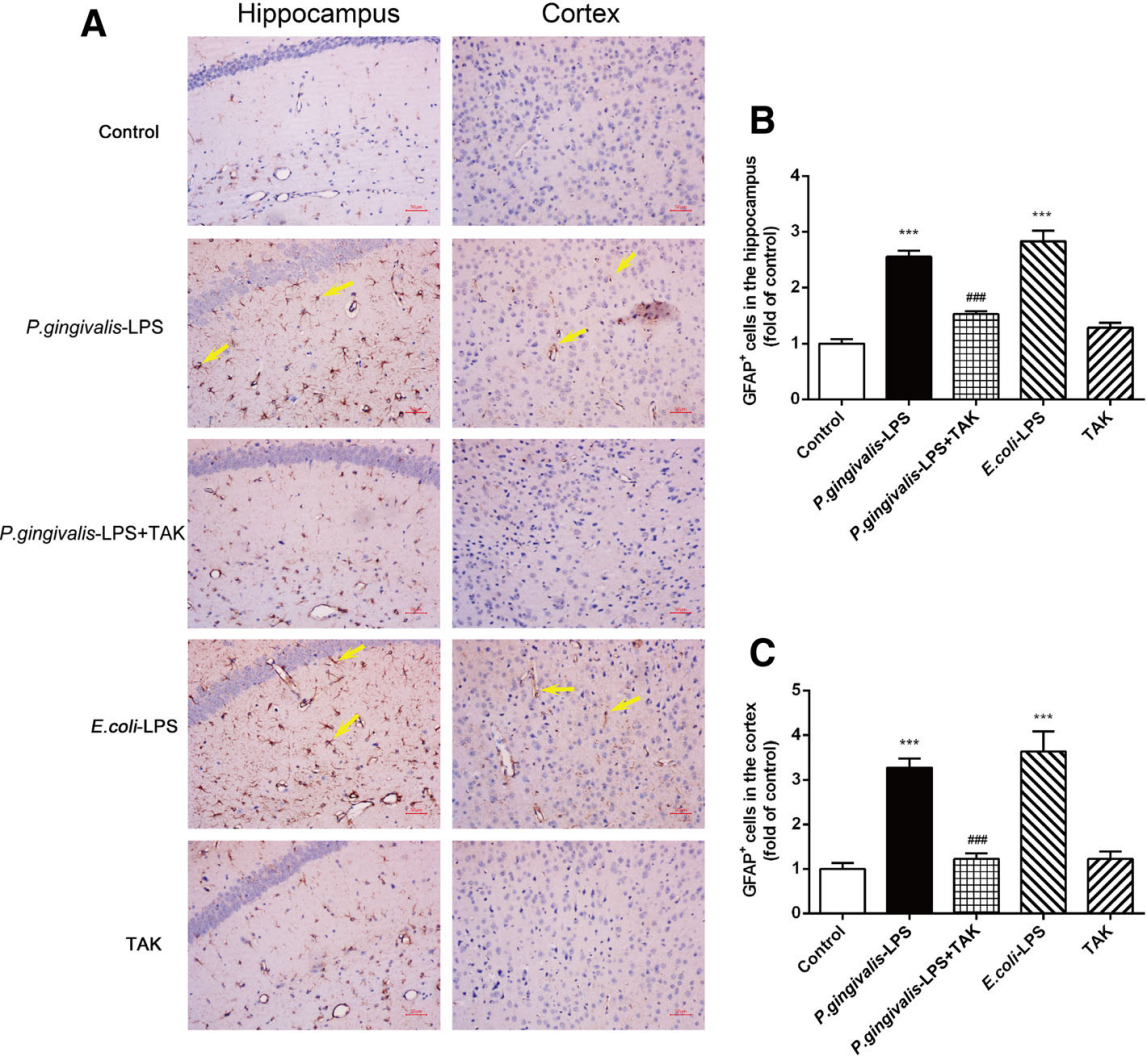
Effects of *P.gingivalis*-LPS on astrocytes in the hippocampus and cortex. Histopathological analysis of brain sections was performed using immunohistochemistry. Astrocytes were visualized with the glial fibrillary acidic protein (GFAP) (**a**, arrows). Quantification of GFAP levels in the hippocampus and cortex are shown (**b**, **c**). Activated astrocytes were significantly increased following *P.gingivalis*-LPS administration. Activation was attenuated by TAK-242 (bar = 50 μm). Data are presented as mean ± SEM; ***p < 0.001, compared to the control group; ^###^*p* < 0.001, compared to the *P.gingivalis*-LPS group

The activated astrocytes were positively stained with GFAP, and cell volume showed a relative increase with hypertrophy and irregular protrusions. In comparison to the control group, the *P. gingivalis*-LPS group and *E. coli*-LPS group had a greater number of activated astrocytes in both the hippocampus and cortex (Fig. 8a). The number of GFAP-positive cells in the *P. gingivalis*-LPS group and the *E. coli*-LPS group was significantly greater in both the cortex and hippocampus, in comparison to the control group. No significant differences were observed between the *P. gingivalis*-LPS group and *E. coli*-LPS group (Fig. 8b).

The changes observed in the activated microglia and astrocytes in the hippocampus and cortex of the *P. gingivalis*-LPS group were significantly prevented by TAK-242. These findings indicate that inflammation might play an important role in the brain and could be associated with Toll-like receptors.

### Effects of *P. gingivalis*-LPS on mRNA and protein expression of inflammatory cytokines

**Fig. 9:**
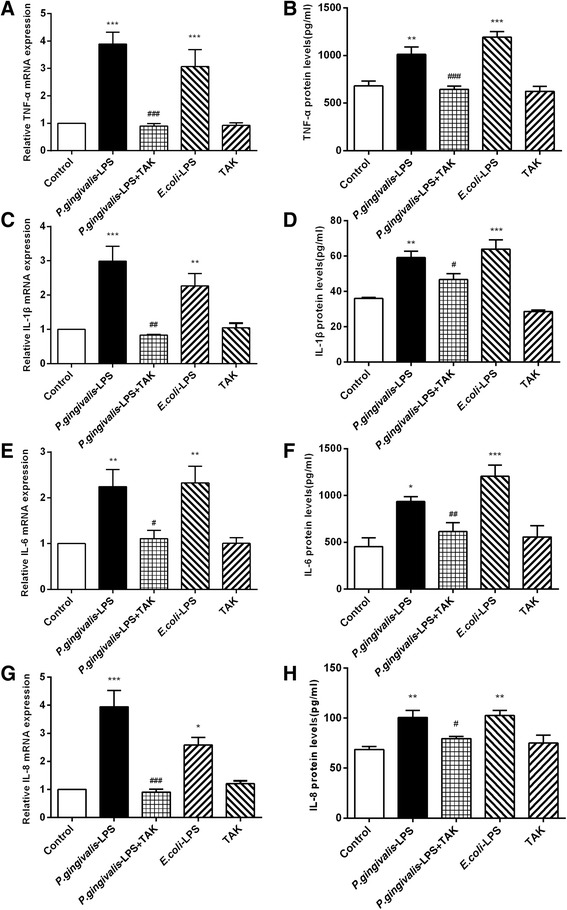
Effects of *P.gingivalis*-LPS on mRNA and protein expression of inflammatory cytokines. RT-PCR and ELISA were performed to detect mRNA (**a**, **c**, **e**, **g**), and protein (**b**, **d**, **f**, **h**) levels of inflammatory cytokines. Administration of *P.gingivalis*-LPS induced high expression of inflammatory factors on genes and proteins, in comparison to the control group, whereas these changes were reversed by TAK-242. Data are presented as mean ± SEM; *p < 0.05, **p < 0.01,  and ***p < 0.001, compared to the control group; ^#^*p* < 0.05, ^##^*p* < 0.01, and ^###^*p* < 0.001, compared to the *P.gingivalis*-LPS group

The RT-PCR assays were performed for mRNA expression of TNF-α, IL-1β, IL-6, and IL-8 genes. The *P. gingivalis*-LPS group showed an approximately fourfold increase in TNF-α mRNA expression, threefold increase in IL-1β mRNA expression, twofold increase in IL-6 mRNA expression, and fourfold increase in IL-8 mRNA expression, in comparison to the control group. The *P. gingivalis*-LPS plus TAK group showed reduced TNF-α, IL-1β, IL-6, and IL-8 mRNA expression in comparison to the *P. gingivalis*-LPS group. No significant differences were observed between the *P. gingivalis*-LPS group and *E. coli*-LPS group (Fig. 9a, c, e, g).

The concentration levels of TNF-α, IL-1β, IL-6, and IL-8 were detected by ELISA. The expression of TNF-α, IL-1β, IL-6, and IL-8 proteins in the *P. gingivalis*-LPS group was significantly higher than that in the control group. In comparison to the *P. gingivalis*-LPS group, the *P. gingivalis*-LPS plus TAK group showed reduced expression of TNF-α, IL-1β, IL-6, and IL-8 proteins. No significant differences were observed between the *P. gingivalis*-LPS group and *E. coli*-LPS group (Fig. 9b, d, f, h).

High expression of inflammatory factors on genes and proteins was stimulated by *P. gingivalis*-LPS. These findings were indicative of neuronal inflammation in the brain. These effects, however, were significantly prevented by TAK-242.

### Effects of *P. gingivalis*-LPS on mRNA expression of TLR2, TLR3, TLR4, and CD14

**Fig. 10:**
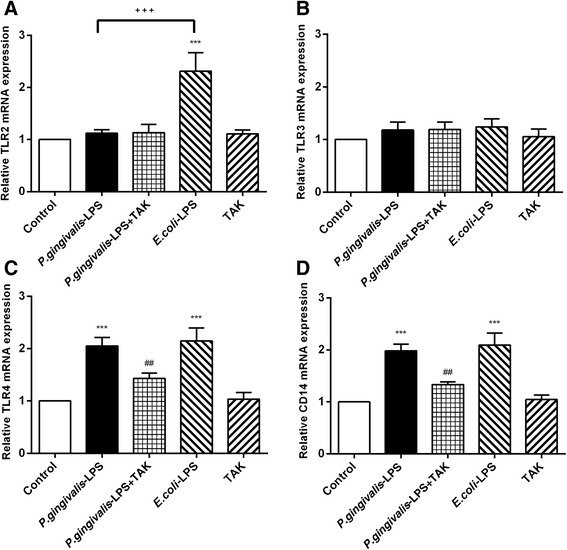
Effects of *P.gingivalis*-LPS on mRNA expression of TLR2, TLR3, TLR4, and CD14. The RT-PCR was performed to detect mRNA levels of TLR2, TLR3, TLR4, and CD14 (**a**–**d**). The mRNA expression of TLR4 and CD14 was upregulated by *P.gingivalis*-LPS; however, similar effects were not observed in TLR2 and TLR3. Data are presented as mean ± SEM; ***p < 0.001, compared to the control group; ^##^*p* < 0.01, compared with the *P.gingivalis*-LPS group; ^+++^*p* < 0.001, *P.gingivalis*-LPS group compared with the *E.coli*-LPS group

The RT-PCR assays were performed for TLR2, TLR3, TLR4, and CD14 genes. The *P. gingivalis*-LPS group induced an approximately twofold increase in TLR4 and CD14 mRNA expression, in comparison to the control group. However, no significant differences were observed in TLR2 and TLR3 mRNA expression in comparison to the control group (Fig. 10a, b). The *P. gingivalis*-LPS plus TAK group showed reduced TLR4 and CD14 mRNA expression in comparison to the *P. gingivalis*-LPS group. No significant differences in TLR4 and CD14 mRNA expression were observed between the *P. gingivalis*-LPS group and *E. coli*-LPS group (Fig. 10c, d). The *E. coli*-LPS group showed an approximately 2.5-fold increase in TLR2 mRNA expression in comparison to the control group, whereas no significant differences were observed between the *P. gingivalis*-LPS group and control group (Fig. 10a). High expression of TLR4 and CD14 genes was promoted by *P. gingivalis*-LPS. The changes induced by *P. gingivalis*-LPS were significantly prevented by TAK-242.

### *P. gingivalis*-LPS-induced neuroinflammatory processes via the TLR4/NF-κB signaling pathway

**Fig. 11:**
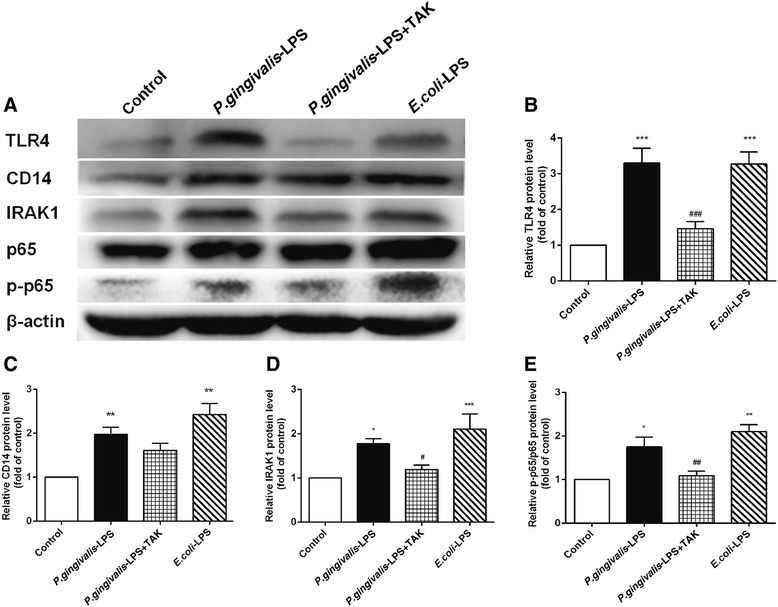
*P.gingivalis*-LPS-induced neuroinflammatory processes via the TLR4/NF-κB signaling pathway. Expression of CD14, TLR4, IRAK1, p65, and p-p65 was measured by western blot analysis (**a**). Graphs show the semiquantitative analysis of protein levels (**b**–**e**). Expression of CD14, TLR4, IRAK1, and p-p65/p65 proteins was upregulated by *P.gingivalis*-LPS. High expression of these proteins (TLR4, IRAK1, and p-p65/p65) was effectively inhibited by TAK-242. Data are presented as mean ± SEM; *p < 0.05, **p < 0.01, ***p < 0.001, compared to the control group; ^#^*p* < 0.05, ^##^*p* < 0.01, ^###^*p* < 0.001, compared to the *P.gingivalis*-LPS group

As shown in Fig. 11, western blot analysis was performed to study the underlying mechanism of *P. gingivalis*-LPS-induced neuroinflammation. We detected elevated protein expression of TLR4, CD14, IRAK1, and p-p65/p65 in *P. gingivalis*-LPS-treated and *E. coli*-LPS-treated mice, in comparison to the control mice. The elevated expression of TLR4, IRAK1, and p-p65/p65 proteins induced by *P. gingivalis*-LPS was attenuated by TAK-242 (Fig. 11b, d, e). Overall, these results provide evidence for the involvement of *P. gingivalis*-LPS in the downstream signal processing of the TLR4/CD14 receptor, through IRAK1 signal processing, and the final activation of the NF-κB signaling pathway.

## Discussion

In the present study, behavioral experiments demonstrated that *P. gingivalis*-LPS could induce cognitive impairment. Immunohistochemistry showed that *P. gingivalis*-LPS significantly activated microglia and astrocytes in the cortex and hippocampus. In addition, the expression of inflammatory cytokines was evidently upregulated by *P. gingivalis*-LPS. Further evaluation indicated that *P. gingivalis*-LPS activated the TLR4/NF-κB signaling pathway, including the TLR4/CD14 receptor, IRAK1, NF-κB, and cytokines. These changes were all effectively prevented by the TLR4 inhibitor TAK-242. To our knowledge, the present study was the first to demonstrate the relationship between periodontal pathogens, such as *P. gingivalis*-derived LPS and cognitive impairment.

Previous studies have demonstrated that *E. coli*-LPS could lead to learning and memory impairment [20, 21]. In the present study, although the administration of either *P. gingivalis*-LPS or *E. coli*-LPS was shown to impair spatial learning and memory in the MWM test, no significant differences were observed between the effects of the two LPS species. Passive avoidance trials illustrated that the ability of mice to respond to passive avoidance memory could be attenuated by *P. gingivalis*-LPS.

Inflammation is known to play an important role in the development and progression of dementia and can damage nerve cells, thereby leading to neuronal apoptosis and cognitive dysfunction [22, 23]. A downstream neuronal inflammatory cascade occurs, and the pathological manifestations of neuroinflammation include the activation of microglia and astrocytes and high expression of inflammatory factors [24, 25].

Microglia play an important role in the inflammatory response. They are the resident macrophages in the brain that regulate its pathological and regenerative processes by producing various molecules, including both neurotrophic and neurotoxic factors. Microglia are important sources of proinflammatory and oxidative stress factors, such as tumor necrosis factor, nitric oxide, interleukins, and other neurotoxic substances [26, 27]. Previous studies have reported that *E. coli*-LPS could stimulate microglial activation and increased the release of inflammatory cytokines [23, 28, 29]. The present study showed that microglia activated by *P. gingivalis*-LPS in the cortex and hippocampus could produce inflammatory cytokines and thereby induce neuroinflammation.

Under pathological conditions, astrocytes change from a resting state to an activated state, and their activation has a cascading effect. Excessive activation of astrocytes can produce toxic effects on neurons. The GFAP is recognized as a characteristic marker of astrocytes [30–32]. Studies conducted by Dihné et al. and Fiedorowicz et al. indicate that activated astrocytes are capable of producing immune inflammatory lesions and cytotoxic substances that can cause tissue damage [33, 34]. The results of the present study showed that astrocytes were significantly activated by *P. gingivalis*-LPS, which may have had toxic effects on neurons.

The inflammatory response is a common observation in the brain tissue of patients with dementia, and neuroinflammation typically occurs before the deposition of amyloid-β (Aβ) [22]. The results of the present study showed that the relative levels of inflammatory cytokines (TNF-α, IL-1β, IL-6, and IL-8) and associated genes and proteins in the *P. gingivalis*-LPS group were significantly increased. Furthermore, these changes were effectively prevented by TAK-242. This suggests that *P. gingivalis*-LPS might stimulate the brain to secrete increased levels of inflammatory cytokines via TLR4 activation and thereby induce neuroinflammation.

Previous studies have produced conflicting results with respect to the *P. gingivalis*-LPS-induced signaling pathway. Reports have shown that TLR4 in conjunction with CD14 plays an important role in *P. gingivalis*-LPS signaling [35–38]. However, other reports demonstrate that *P. gingivalis*-LPS signaling occurs primarily via TLR2, but not TLR4 [39, 40]. The results of the present study showed that *P. gingivalis*-LPS increased TLR4 and CD14 mRNA expression. In addition, TAK-242, a selective inhibitor of TLR4 signaling [41, 42], inhibited the high mRNA expression of TLR4 and CD14 caused by *P. gingivalis*-LPS. These results suggest that TLR4 was involved in *P. gingivalis*-LPS signaling. Moreover, the expression of TLR4, IRAK1, and p-p65/p65 proteins was upregulated by *P. gingivalis*-LPS. These findings evidently support the downstream signal processing of *P. gingivalis*-LPS and its association with the TLR4/CD14 receptor, via IRAK1 signal processing, and the final activation of the NF-κB signaling pathway.

## Conclusions

In summary, to our knowledge, the present study is the first to demonstrate the effects of *P. gingivalis*-LPS on cognitive function in vivo and the associated underlying mechanisms. We found that *P. gingivalis*-LPS can lead to learning and memory impairment in mice. Inflammation plays an important role in cognitive impairment, which is mediated by activation of the TLR4 signaling pathway. Our findings also reveal that endotoxins of periodontal pathogens could represent a risk factor for cognitive disorders. Further progress in studies of the relationship between CP and cognitive diseases could usher in novel changes in the treatment and prevention of cognitive disorders.

## Abbreviations

ANOVA: Analysis of variance
BCA: Bicinchoninic acid
CP: Chronic periodontitis
DAB: 3,3′-Diaminobenzidine
ELISA: Enzyme-linked immunosorbent assay
GFAP: Glial fibrillary acidic protein
LPS: Lipopolysaccharide
MWM: Morris water maze
OFT: Open field test
PAT: Passive avoidance test
PBS: Phosphate-buffered saline
PMSF: Phenylmethylsulfonyl fluoride
PVDF: Polyvinylidene difluoride
RIPA: Radioimmunoprecipitation assay
SDS: Sodium dodecyl sulfate
SEM: Standard error of the mean
